# Effectiveness of Systematic Health Education Model for Type 2 Diabetes Patients

**DOI:** 10.1155/2018/6530607

**Published:** 2018-07-25

**Authors:** Yongwen Zhang, Lanfang Chu

**Affiliations:** ^1^Department of Endocrinology, Nanjing Integrated Chinese and Western Medicine Hospital Affiliated to Nanjing University of Chinese Medicine, Nanjing 210014, China; ^2^Department of Integrated Traditional Chinese and Western Medicine, Nanjing General Hospital of Nanjing Military Command, Nanjing 210012, China

## Abstract

**Background:**

Health education is considered to be essential in the overall care of patients with type 2 diabetes mellitus (T2DM); systematic health education integrates individual education not only during hospitalization but also extended care outside of a hospital. To test effectiveness of the systematic health education model for T2DM, we conducted a randomized study with a control group among patients with T2DM living in Nanjing, China.

**Methods:**

998 eligible patients completed the enrollment and were randomized to systematic health education model and conventional model groups (498 and 500 patients, resp.). The systematic health education model was based on the following aspects: image education, visitation of the exhibition hall, dissemination of educational materials, individualized medical nutrition therapy and exercise programs, WeChat group and regular health lectures, evaluation of complications, lifestyle modification, systematic treatment scheme, self-monitoring of glycemic control, monthly evaluation of the therapeutic effect, proposed improvement measures, and individualized follow-up scheme. The main outcome measures were glycated hemoglobin A1c (HbA1c), blood pressure, body mass index (BMI), and lipids during the 2-year follow-up.

**Results:**

The systematic health education model led to a favorable variation in HbA1c, LDL cholesterol, and systolic blood pressure (SBP) (*P* < 0.05). After adjusted analysis, the HbA1c decreased by 0.67% (*P* < 0.01) in the systematic health education model, SBP decreased by 10.83 mmHg (*P* < 0.01), and the level of diastolic blood pressure (DBP), HDL cholesterol, and total cholesterol decreased slightly and was not significant. The BMI did not change significantly during the study in either of the two groups.

**Conclusions:**

The systematic health education model is a useful method in the treatment of T2DM because it contributes to decrease in HbA1c, LDL cholesterol, and SBP levels, as well as helps in increasing the compliance with the control criteria, except for DBP and BMI.

## 1. Background

Over the last 20 years, a conceptual transformation in the principles of management of type 2 diabetes mellitus (T2DM) has occurred. Treatment for T2DM involves controlling glycemic and metabolic levels, helping patients and their families to adapt to their situation on a psychosocial level, preventing serious or chronic complications, decreasing health care costs, ensuring that medications are taken on a regular basis, and, in particular, promoting a change in lifestyle [1]. In 2011, our hospital integrated diabetes education in the hospital's outpatient department. We incorporated several new methods into the health education model, including restricted diet with a plate, WeChat group, and individualized follow-up scheme. Systematic health education is one of the different educational models that focus on factors influencing health behavior in the whole course of disease. It is based on the relationship between the health educator and the patient, which is particularly appropriate for chronic diseases. Systematic health education integrates individual education not only during hospitalization but also extended care outside of a hospital. There have been very few long-term studies, with randomized controls, on the effects of systematic health education in T2DM. To test effectiveness of the systematic health education model for T2DM, we conducted a randomized study with a control group among patients with T2DM living in Nanjing, China. Therefore, the aim of this study was to assess the effectiveness of the systematic health education on the changes in lipids, HbA1c, body mass index (BMI), and blood pressure (BP) in patients with T2DM over 2 years.

## 2. Methods

### 2.1. Patients

**Figure 1:**
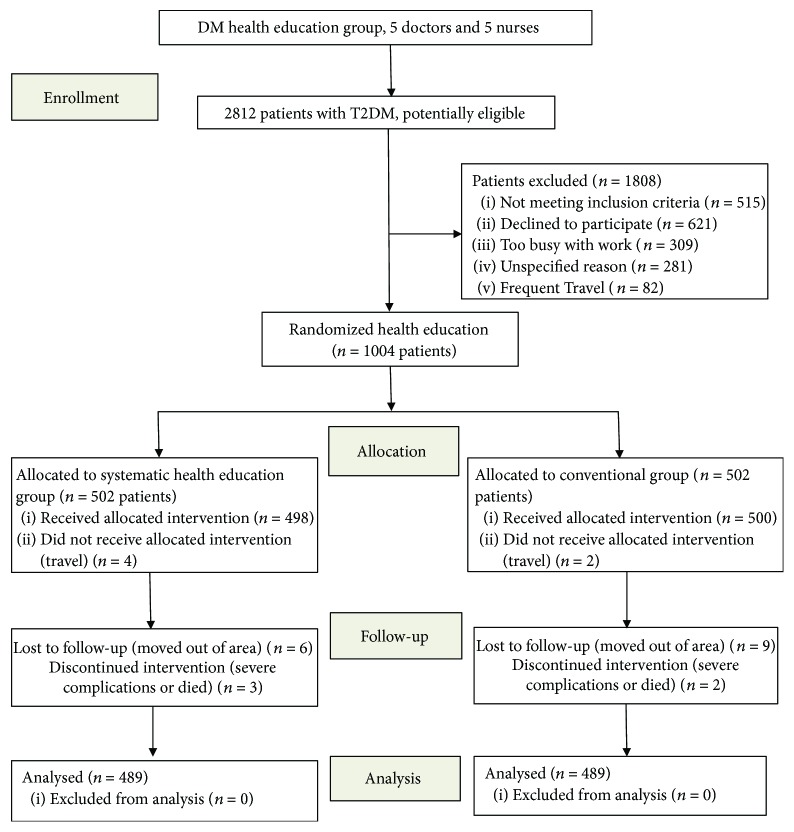
Flow diagram of participants.

The participants were randomized by selection from the hospital's inpatient and outpatient wards. The research group was comprised of 10 persons including endocrinologists, nurses, dietitians, exercise specialists, behavioral therapists, and pharmacists. The study was approved by the ethics committee of the Nanjing Integrated Chinese and Western Medicine Hospital Affiliated to Nanjing University of Chinese Medicine. The inclusion criteria for this study included the following: male or female older than 18 years and less than 65 years old, diagnosis of T2DM, and at least one treatment prior to the study. The exclusion criteria were patients with type I diabetes or gestational diabetes, patients with life expectancy of less than 2 years, patients involved in trials, and patients who declined to participate. Patients meeting the criteria for inclusion and not meeting the exclusion criteria were included after signing an informed consent form. All participants were incentivized with a blood glucose meter.  Figure 1 shows the patient recruitment process.

### 2.2. Methods

This study was conducted from March 2011 to November 2017. Visits in both groups including the usual care and systematic care are based on the conventional or systematic health education models, respectively. The systematic intervention which was developed specifically for this study contained nine components: a low-literacy color booklet, a motivational video, restricted diet with a plate, WeChat group and regular health lectures, targeted treatment, group medical visits, improvement of the uptake and maintenance of medication regimes, lifestyle interventions, and self-management educational interventions. Subjects in the control group were disseminated self-educational materials and had face-to-face lectures quarterly. The Diabetes Health Education Group includes the leading organizations of health professionals and volunteers; the volunteers help promote and disseminate educational materials on diabetes control and prevention. The committee meeting, with the duration of less than half an hour, is held twice per year if any changes are needed in program operations and to review the findings of process evaluation activities. Personal benefit of the therapeutic patient education practice for health care professionals, emotional dimension, holistic and interdisciplinary approach to the patient, the professionalizing nature of therapeutic patient education, educational relationship between the patient and caregiver, and ethical relationship are included in the diabetes educators' clinical training programs [2].

### 2.3. Patient Education Programs

The objectives of health education are to support informed decision-making, problem-solving, active collaboration with the health education team, and self-care behaviors as well as to improve clinical outcomes, quality of life, and health status. These objectives are accomplished through systematic health education in a group provided by health educators every month for over 2 years. A total of 2812 individuals were invited to participate in the study. The reasons for declining participation included frequent travel, too busy with work, lack of interest, and unable to be contacted by WeChat or phone.

The data gathered were sociodemographic variables, dietary habits, alcohol consumption, tobacco consumption, physical activity, foot self-care, self-monitoring of capillary glycemia, and medication adherence [3], along with associated morbidity such as obesity; hypertension (HTN); dyslipidemia; ischemic cardiopathies including acute myocardial infarction (AMI), angina, and cerebrovascular accident (CVA); and DM complications (neuropathy, microvascular, retinopathy, and macrovascular). In follow-up visits, data were collected on various parameters (HbA1c, total cholesterol, low-density lipoprotein (LDL) cholesterol, systolic blood pressure (SBP), diastolic blood pressure (DBP), high-density lipoprotein (HDL) cholesterol, and BMI). The systematic health education model was based on the following aspects:

#### 2.3.1. Image Education

All patients were provided with video on DM and HTN, which is based on audiovisual techniques to create wider awareness about the importance of diabetes. Image education can offer patients more details about DM pathogenesis, harmfulness, complications, and treatment, thereby increasing knowledge regarding the importance of controlling risk factors. Sometimes, family members would participate in image education; all were welcome.

#### 2.3.2. Visitation of the Exhibition Hall

With on-site visits, patients can acquire general knowledge of medication effects on the body, food model, alternative medication options, self-injection of insulin, drug prescription instructions, warning signs of hypoglycemia, and carbohydrate-counting technique.

#### 2.3.3. Dissemination of Educational Materials

In order to improve diabetes self-management and to be an active participant in the whole process, a low-literacy color booklet was made by our group. The patients in the group received the paper booklet containing the question-explanation materials on diabetes and therapy targets.

#### 2.3.4. Individualized Medical Nutrition Therapy

**Figure 2:**
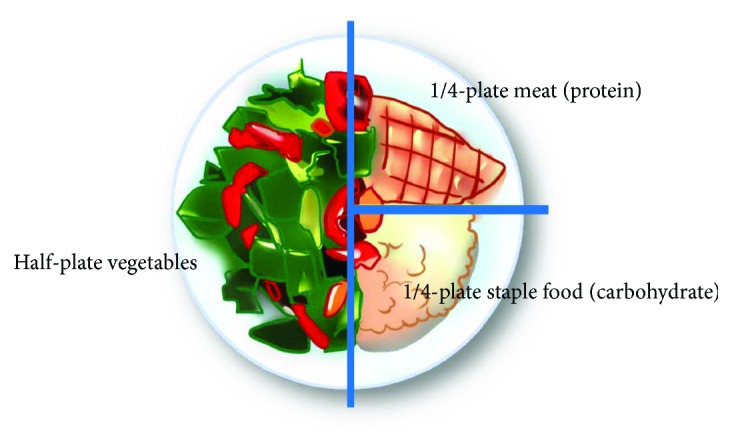
Restricted diet with a plate.

In a comprehensive and individually negotiated nutrition program, each patient's preferences, cultural background, and circumstances as well as the overall treatment program are considered. Because of the complexity of the medical and nutritional issues for most patients, our team recommended a simplified scheme called the “restricted diet with a plate” (Figure 2), which consists of three parts: half-plate vegetables, 1/4-plate staple food (carbohydrate), and 1/4-plate meat (protein).

#### 2.3.5. Individualized Exercise Programs

Exercises include walking, jogging, cycling, swimming, Taijiquan, gymnastics, badminton, yangko dancing, and square dancing. Physical exercise goals, methods, frequencies, and intensities must be negotiated with patients with great sensitivity to recognizing barriers and helping patients find solutions. Patients who develop symptoms of coronary ischemia should be referred for further evaluation and treatment.

#### 2.3.6. WeChat Group and Regular Health Lectures

Face-to-face lectures were the most common delivery format, and the frequency of educational lectures was monthly. The WeChat group provides the means of giving patients better health education. Unless results are generally within agreed target ranges, the patients should be reviewed and communicated regularly with the health education team by WeChat or at an interim visit to trigger changes in therapy as the need arises. The primary roles of the lectures are to supply guidance in goal setting to manage the risk of complications, sharing experiences and techniques to overcome barriers, suggest strategies for achieving goals, help screen for complications, and provide training in skills.

#### 2.3.7. Evaluation of Complications

The major role of the team is to screen for complications (neuropathy, vascular disease, retinopathy, and nephropathy) and discover ways for patients to be able to exercise safely [1]. The group provided guidance in screening for complications, develop treatment plans, evaluate progress in meeting treatment goals, and help develop strategies for achieving treatment goals and avoiding complications.

#### 2.3.8. Lifestyle Modification

Lifestyle therapy consists of weight loss; reduced saturated fat, trans fat, and cholesterol intake; reduced sodium and increased potassium intake; moderation of alcohol intake; quitting smoking; and increased physical activity. The components of lifestyle intervention include exercise recommendations, medical nutrition counseling, and comprehensive diabetes education with the purpose of changing the paradigm of care in diabetes from provider focused to patient focused [1].

#### 2.3.9. Systematic Treatment Scheme

In order to successfully implement this process, the patient must participate fully in the development of a treatment plan, commit to the principles of self-care, make ongoing decisions regarding self-care from day to day, communicate honestly and with sufficient frequency with the team, reduce the daily intake of bread, increase physical activity, eat fewer times a day, self-monitor their blood glucose, improve medication adherence, and improve skills for insulin treatment.

#### 2.3.10. Self-Monitoring of Glycemic Control

Patients were encouraged to monitor their blood glucose, record values, and bring a record book to appointments. Many patients faithfully perform daily self-monitoring of blood glucose, record the results as instructed, and discuss them with the health education team. The patients should communicate with the team when goals are not achieved or when problems or barriers are encountered.

#### 2.3.11. Monthly Evaluation of the Therapeutic Effect

**Figure 3:**
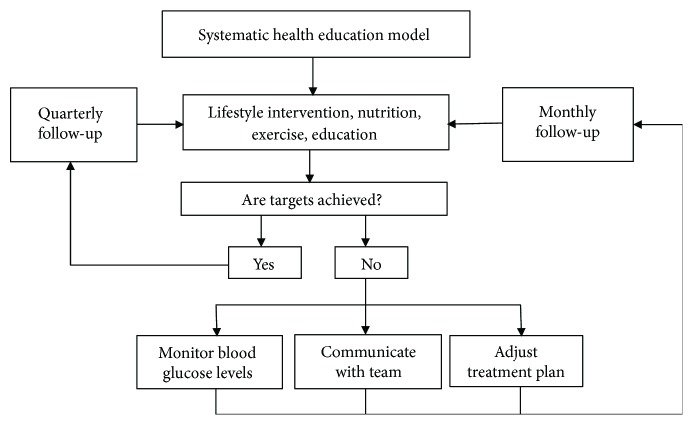
Flow diagram of systematic health education model.

Reinforcing treatment, goal setting to promote health, and problem-solving for daily living are necessary; the doctors provide patients with clear control objectives such as HbA1c, LDL cholesterol, HDL cholesterol, total cholesterol, and blood pressure. Patients need to be encouraged to integrate lifestyle intervention into daily life, participating more actively in the process. The patients should communicate honestly and with sufficient frequency with the team.  Figure 3 shows the flow diagram of evaluating the therapeutic effect.

#### 2.3.12. Proposed Improvement Measures

A further improved plan was proposed based on the therapeutic efficacy, blood glucose, complications, and adverse reactions. A repeated diet history and additional modest changes negotiated every few weeks to months by the group allow assessment of whether previously agreed to changes were enacted, allow reinforcement of the importance of diet efforts, and allow slow enticement of patients into more healthful dietary choices (Figure 3).

#### 2.3.13. Individualized Follow-Up Scheme

Regular follow-up must be an integral component of its long-term management. The individual follow-up scheme contained follow-up time, risk factors, foot care, self-injection of insulin, warning signs of hypoglycemia, questions on diabetes attitude, and hypoglycemic coping measures. These programs should periodically reinforce behavior change and long-term sustainability.

### 2.4. Statistical Analysis

A descriptive analysis was performed for each variable, involving the frequencies with confidence intervals of 95% (95% CI) for the qualitative variables and mean and SD for the quantitative variables. Student's *t*-test or its nonparametric equivalent was used for paired data, McNemar's test was used for paired data, and Pearson's *χ*^2^ test was used for the qualitative variables. The change was calculated in systematic health education and conventional groups for the following variables: HbA1c, HDL cholesterol, LDL cholesterol, total cholesterol, SBP, and DBP. The effect of the systematic health education model was performed for these variables using the following formula: mean value of the change in systematic health education model − mean value of the change in conventional education model. The ANCOVA was used to determine the adjusted effect of the systematic health education model. If the *P* value was less than 0.05, it indicated that the difference of the feature between two models was statistically significant. Analyses were performed on the intention-to-treat principle; that is, all subjects in the study were assumed to have received a full dose of the intervention.

## 3. Results

Of 2812 potentially eligible patients, 1004 (35.7%) requested to enroll. Among these, 998 (99.4%) completed the enrollment and were randomized to systematic health education model and conventional model groups (498 and 500 patients, resp.) (Figure 1). The two groups studied according to the type of health education model were observed to be homogeneous in terms of age, T2DM duration, and gender. The baseline clinical characteristics of the two groups, adherence to medication, distribution of morbidity, adherence to diet, and chronic complications are shown in Table 1. Demographic characteristics of randomized patients were similar between groups; significant differences were not observed between the groups (Table 1).

The systematic health education model led to a favorable variation in HbA1c, LDL cholesterol, and SBP (*P* < 0.05); the conventional model achieve an improvement in all control criteria, but statistically significant differences were not observed (Table 2). The nonadjusted effect of systematic health education model on the changes in parameters was greater for LDL, HbA1c, and SBP; this showed significant differences between the two models (*P* < 0.05). After adjusted analysis, the HbA1c decreased by 0.67% (*P* < 0.01) in the systematic health education model. Furthermore, SBP decreased by 10.83 mmHg (*P* < 0.01), and the level of DBP, HDL, and total cholesterol decreased slightly and was not significant. The BMI did not change significantly during the study in either of the two groups, and the adjusted effect of systematic health education model was −0.23 (Table 2).

However, after 2 years of follow-up, the systematic health education model was better than the conventional model in percentage of subjects on-target for cardiovascular risk factors: LDL cholesterol < 100 mg/dl (*P* = 0.02), HbA1c < 7% (*P* < 0.01), BP control (<130/80 mmHg) (*P* = 0.03), SBP < 130 mmHg (*P* = 0.03), and global control (metabolic and BP) (*P* < 0.01). Nevertheless, it was not significant for the criteria DBP < 80 mmHg and BMI < 25 kg/m^2^ (Table 3).

## 4. Discussion

Diabetes is a chronic disease, and health educators have almost no control over the extent to which patients adhere to the day-to-day treatment plan. As defined by the ADA, diabetes self-management education is the process of providing to the person with diabetes knowledge and skills needed to perform self-care, manage crises, and make lifestyle changes [4–6]. For long-term success, diabetes health education is critical. The National Diabetes Education Program (NDEP) was established to translate findings from diabetes research studies into clinical and public health practice [7]. To achieve this task, patients and providers work together in a long-term, ongoing process. Although there are only limited studies, they do provide support for the concept that diabetes education can be cost-effective and can improve outcomes [8]. A team of educators is usually required to fully implement the process of diabetes health education, because the needed range of expertise is broad and the amount of information that needs to be exchanged is large. For health education to be most effective, trust, mutual respect, and communication are critical. However, in many communities, the full benefit of consultation and ongoing care with diabetes educators, nurses, dietitians, pharmacists, or others is not achieved because of overly hierarchic approaches to education [1].

The reduction in HbA1c (−0.67%) observed in our study is higher than that achieved by other health education strategies. The study carried out by Salinero-Fort et al. showed a reduction in HbA1c of −0.18% (*P* = 0.01) after a 2-year follow-up period [9]. The meta-analysis by Norris et al. showed a decreased HbA1c from the baseline of −0.26% (95% CI; −0.05 to −0.48) at ≥4 months [10]. In different pharmacological intervention studies, a decrease in HbA1c levels has shown a reduction in microvascular and macrovascular complications after long-term follow-up [11, 12]. In the United Kingdom Prospective Diabetes Study (UKPDS), patients were treated with diet and exercise for 3 months, with an average reduction in HbA1c from approximately 9% to 7%. Associated with this improvement in glycemic control, there was a reduction in the risk of microvascular complications in the group receiving intensive treatment [13]. These results as well as those obtained in our study suggest that pharmacological treatments need to be complemented with booklets, exercise, and lifestyle-modifying strategies [9]. In aggregate, our study provides evidence that systematic health education can generate sustained improvements in BP, glucose control, and metabolic control. The control group also sustained substantial reductions in HbA1c (−0.38%), but there was no significant difference (*P* = 0.57). This is showing that booklets and face-to-face lectures can significantly improve HbA1c over 24 months [14, 15].

The reduction in SBP (10.83 mmHg) obtained in our study is greater than that found in studies carried out by Hiss et al. and Shibayama et al. [16, 17]. The meta-analysis carried out by Duke et al. pointed out that the mean adjusted reduction was 1.86 mmHg after 12–18 months [18]. The study showed a reduction of 7% in the risk of mortality owing to cardiovascular disease and 10% in the risk of mortality owing to ictus with every 2 mmHg decrease in SBP [19]. The difficulty in reducing the BMI and total cholesterol may be explained by the improvement of living standards, changes in dietary structure, and reduction of physical activity. Trento et al. demonstrated that BMI decreased over 5 years among group care (−1.4, 95% CI; −2.0 to −0.7), but there was no statistically significant difference observed (*P* = 0.067) [20]. Furthermore, a difficulty in reducing the BMI was mentioned in the study by Scain et al., which also showed no differences when compared with normal care, although the BMI did decrease significantly when compared with the baseline [21].

The increase obtained in the proportion of patients within HbA1c, BP control, and global control after the application of systematic health education model is relevant and suggests that there is a need to complement pharmacological treatments with systematic health education. In this study, we found that resources for T2DM health education were not adequate; to make these programs efficient, other resources such as face-to-face lectures, diet control, pamphlets, booklets, and video should be made available. There should be a multisystem approach towards making these resources available. Trento et al. developed a model to deliver diabetes care as group education sessions, which improves clinical outcomes, patients' quality of life, and clinicians' satisfaction while optimizing use of the typically limited resources of busy clinics [22]. The effect of systematic health education on the patients' quality of life needs to be studied further.

The face-to-face method is still the common measure for conducting health education for T2DM [23]. However, with the increasing number of diabetic patients, this method is difficult for doctors who have so many other responsibilities. To reduce the time spent on health education, it is essential to provide health education materials with pamphlets and booklets used as good references by educated patients. Although the use of audio is helpful in disseminating information, they were scarce in health education. All health education should be provided with booklets and video as a matter of urgency particularly in those that do not have enough T2DM health educators. Therefore, the provision of health education materials is a matter of urgency.

Our study has several strengths, including few losses, the novelty of the restricted diet with a plate, and patients merely requiring a mobile device. Our findings must also be considered in the context of several limitations. The most important limitation is it being a nonblind trial, with the possibility of bias during study. Enrollment in the study required a mobile device; this may limit its generalizability, as may the predominantly young patients from which were recruited. A multicenter controlled trial (4 years) by Trento et al. showed that patients in group care had lower A1c, total cholesterol, LDL cholesterol, triglycerides, systolic and diastolic blood pressure, BMI, and serum creatinine and higher HDL cholesterol (*P* < 0.001, for all) than control subjects receiving individual care [22]. We do not have outcomes beyond 2 years, so we cannot determine the degree to which the changes in HbA1c, metabolic control, and blood pressure are durable.

## 5. Conclusions

As a result of all the abovementioned factors, it can be concluded that the systematic health education model is a useful method in the treatment of T2DM, because it contributes to decrease in HbA1c, LDL cholesterol, and SBP levels, as well as helps in increasing the compliance with the control criteria, except for DBP and BMI. Because health education can result in cost savings and improved outcomes, health education should be covered by insurance and other payers. Competent health educators in China are few, which makes it difficult to cover all DM patients. To overcome this shortage, educators who care for DM must be given a systematic health education training to increase awareness of the seriousness of diabetes, its risk factors, and strategies for preventing diabetes and its complications among groups at risk [24].

## Figures and Tables

**Table 1:** Baseline characteristics of participants.

	SHEM (*n* = 489)	Control (*n* = 489)	*P* value
Age (years)	50.8 (14.3)	52.6 (13.2)	0.76
Female (% (95% CI))	50.7 (43.2–58.2)	48.9 (40.3–57.5)	0.57
DM duration (years)	10.5 (10.0)	11.1 (8.8)	0.88
Smoker (% (95% CI))	9.6 (5.3–13.9)	11.9 (4.6–19.2)	0.26
Number of cigarettes/day	11.3 (6.6)	10.9 (7.3)	0.89
Alcohol/week (units)	4.4 (3.3)	6.0 (3.2)	0.24
Exercise (hours/week)	7.5 (2.5)	8.1 (2.3)	0.53
Compliance with diet (% (95% CI))	37.0 (32.2–41.8)	40.5 (36.3–44.7)	0.27
Self-control (% (95% CI))	33.5 (31.8–35.2)	30.1 (28.8–31.4)	0.24
Foot care (% (95% CI))	23.1 (19.5–26.7)	20.9 (17.7–24.1)	0.40
Therapeutic compliance (% (95% CI))	49.3 (45.1–53.5)	52.6 (49.2–56.0)	0.31

History of (% (95% CI))			
Hypertension	66.1 (60.6–71.6)	64.9 (60.3–69.5)	0.46
Stroke	12.1 (8.5–15.7)	9.8 (6.5–13.1)	0.26
AMI	4.5 (2.3–6.7)	5.5 (3.1–7.9)	0.46
CHD angina	20.2 (15.9–24.5)	23.3 (19.1–27.5)	0.25
Dyslipidemia	48.9 (43.4–54.4)	51.3 (45.7–56.9)	0.44
Neuropathy	14.1 (10.3–17.9)	10.8 (7.3–14.3)	0.12
Retinopathy	16.0 (11.5–20.5)	19.2 (14.1–24.3)	0.18
Nephropathy	7.6 (4.4–10.8)	10.4 (6.9–13.9)	0.12

Biochemical and biological parameters			
HbA1c (%)	7.86 (1.2)	8.15 (1.5)	0.61
Total cholesterol (mg/dl)	212.4 (64.9)	195.6 (62.6)	0.53
LDL cholesterol (mg/dl)	128.8 (9.6)	131.2 (9.5)	0.56
HDL cholesterol (mg/dl)	62.3 (15.9)	61.2 (19.5)	0.87
Systolic blood pressure (mmHg)	137.5 (15.4)	141.7 (15.9)	0.52
Diastolic blood pressure (mmHg)	89.0 (14.9)	84.0 (16.0)	0.44
Body mass index (kg/m^2^)	25.3 (4.5)	26.6 (6.2)	0.58

Values are given as mean (SD) unless otherwise specified. SHEM: systematic health education model; CI: confidence interval; CHD: coronary heart disease; AMI: acute myocardial infarction.

**Table 2:** Mean values (SD) of basal and final parameters.

	SHEM (*n* = 489)	Control (*n* = 489)	Unadjusted SHEM effect (95% CI)	Adjusted SHEM effect (95% CI)
HbA1c (%)				
Basal	7.86 (1.2)	8.15 (1.5)		
Final	6.91 (0.7)	7.78 (1.3)		
Change	−0.95 (0.4)	−0.38 (0.6)	−0.87 (−0.01 to 1.7)	−0.67 (−0.97 to −0.36)
*P* value	0.03	0.57	0.047	<0.01

Total cholesterol (mg/dl)				
Basal	212.4 (64.9)	195.6 (62.6)		
Final	204.5 (68.6)	182.2 (60.1)		
Change	−7.91 (27)	−13.50 (25)	−22.3 (−32.2 to 76.9)	−6.7 (−15.1 to 28.5)
*P* value	0.77	0.60	0.41	0.53

LDL cholesterol (mg/dl)				
Basal	128.8 (9.6)	131.2 (9.5)		
Final	113.8 (14.8)	124.8 (9.2)		
Change	−15.1 (5.1)	−6.33 (3.8)	−11.1 (0.66 to 21.5)	−9.6 (−19.0 to −1.93)
*P* value	<0.01	0.11	0.04	0.046

HDL cholesterol (mg/dl)				
Basal	62.3 (15.9)	61.2 (19.5)		
Final	64.1 (13.1)	65.8 (18.1)		
Change	1.8 (5.9)	4.58 (7.6)	1.67 (−11.7 to 15.0)	2.52 (10.2 to 5.2)
*P* value	0.77	0.56	0.80	0.51

SBP (mmHg)				
Basal	137.5 (15.4)	141.6 (15.9)		
Final	125.0 (8.26)	138.4 (14.1)		
Change	−12.5 (5.1)	−3.25 (6.1)	−13.4 (−3.6 to −23.2)	−10.83 (−16.3 to-5.34)
*P* value	0.02	0.601	<0.01	<0.01

DBP (mmHg)				
Basal	89.0 (14.9)	84.0 (16.0)		
Final	85.8 (12.4)	82.7 (13.8)		
Change	−3.25 (5.6)	−1.33 (6.1)	−3.1 (−14.2 to 8.0)	−1.01 (−3.98 to 1.95)
*P* value	0.57	0.83	0.57	0.48

BMI (kg/m^2^)				
Basal	25.33 (4.5)	26.58 (6.2)		
Final	24.67 (4.3)	25.58 (5.7)		
Change	−0.67 (1.8)	−1.00 (2.4)	−0.92 (−3.4 to 5.2)	−0.23(−0.6 to 1.1)
*P* value	0.71	0.69	0.66	0.57

**Table 3:** Percentage of subjects on-target for cardiovascular risk factors.

Target	Model	Baseline (%)	2 years (%)	*P* value	Change (%)	*P* value
HbA1c (<7%)	Control	37.4	42.7	0.09	+5.3	<0.01
	SHEM	34.6	57.1	<0.01	+22.5	
LDL (<100 mg/dl)	Control	20.0	23.1	0.24	+3.1	0.02
	SHEM	23.3	29.9	0.02	+6.6	
SBP (<130 mmHg)	Control	39.3	42.5	0.30	+3.2	0.03
	SHEM	36.6	49.3	<0.01	+12.7	
DBP (<80 mmHg)	Control	42.7	45.6	0.34	+2.9	0.1
	SHEM	46.4	50.9	0.16	+4.5	
BP control^1^	Control	29.0	34.2	0.09	+5.2	0.03
	SHEM	26.4	41.1	<0.01	+14.7	
Global control^2^	Control	21.9	24.7	0.29	+2.8	<0.01
	SHEM	23.9	38.9	<0.01	+15	
BMI (<25 kg/m^2^)	Control	55.0	57.5	0.44	+2.5	0.38
	SHEM	51.3	54.6	0.31	+3.3	

^1^SBP < 130 mmHg and DBP < 80 mmHg. ^2^Metabolic control and BP control.

## Data Availability

The data used to support the findings of this study are available from the corresponding author upon request.
